# Reference Evapotranspiration Modeling Using New Heuristic Methods

**DOI:** 10.3390/e22050547

**Published:** 2020-05-13

**Authors:** Rana Muhammad Adnan, Zhihuan Chen, Xiaohui Yuan, Ozgur Kisi, Ahmed El-Shafie, Alban Kuriqi, Misbah Ikram

**Affiliations:** 1 College of Hydrology and Water Resources, State Key Laboratory of Hydrology-Water Resources and Hydraulic Engineering, Hohai University, Nanjing 210098, China; rana@hhu.edu.cn (R.M.A.); misbahrana2@gmail.com (M.I.); 2 School of Information Science and Engineering, Wuhan University of Science and Technology, Wuhan 430081, China; 3 School of Hydropower and Information Engineering, Huazhong University of Science & Technology, Wuhan 430074, China; 4 Hubei Provincial Key Laboratory for Operation and Control of Cascaded Hydropower Station, China Three Gorges University, Yichang 443002, China; 5 Department of Civil Engineering, School of Technology, Ilia State University, 0162 Tbilisi, Georgia; ozgur.kisi@iliauni.edu.ge; 6 Department of Civil Engineering, Faculty of Engineering, University Malaya, Kuala Lumpur 50603, Malaysia; elshafie@um.edu.my; 7 CERIS—Civil Engineering Research and Innovation for Sustainability, Instituto Superior Técnico—Universidade de Lisboa, 1049-001 Lisboa, Portugal; alban.kuriqi@tecnico.ulisboa.pt; 8 Department of Irrigation and Drainage, University of Agriculture, Faisalabad 38000, Pakistan

**Keywords:** reference evapotranspiration, temperature input, least square support vector regression, gravitational search algorithm, dynamic evolving neural-fuzzy inference system

## Abstract

The study investigates the potential of two new machine learning methods, least-square support vector regression with a gravitational search algorithm (LSSVR-GSA) and the dynamic evolving neural-fuzzy inference system (DENFIS), for modeling reference evapotranspiration (ETo) using limited data. The results of the new methods are compared with the M5 model tree (M5RT) approach. Previous values of temperature data and extraterrestrial radiation information obtained from three stations, in China, are used as inputs to the models. The estimation exactness of the models is measured by three statistics: root mean square error, mean absolute error, and determination coefficient. According to the results, the temperature or extraterrestrial radiation-based LSSVR-GSA models perform superiorly to the DENFIS and M5RT models in terms of estimating monthly ETo. However, in some cases, a slight difference was found between the LSSVR-GSA and DENFIS methods. The results indicate that better prediction accuracy may be obtained using only extraterrestrial radiation information for all three methods. The prediction accuracy of the models is not generally improved by including periodicity information in the inputs. Using optimum air temperature and extraterrestrial radiation inputs together generally does not increase the accuracy of the applied methods in the estimation of monthly ETo.

## 1. Introduction

For irrigation management, agricultural processes, and hydrological cycles, the reference evapotranspiration (ETo) is considered as one of the fundamental variables that should be accurately estimated [1]. For example, it is essential to adequately estimate the ETo in order to figure out the amount, timing, schedule, and frequency for the agricultural procedure and irrigation management [2]. Additionally, nowadays under the expected increase of water scarcity and the reduction of food production, it is vital to get proper knowledge of the estimation of the ETo at different time increments in order to optimize the management of the available water resources, whether conventional or non-conventional water resources [3,4]. Estimation of ETo is fundamental due to the ETo key role in affecting the hydrological cycle [5]. Accurate estimation of ETo will help in understanding the impacts of human influences on the hydrological cycle and in improving water resource management [6].

Estimation of ETo is a challenging task due to complex interactions between meteorological and site-specific factors [7]. Generally, there is a need to standardize a single robust method to be used at a large scale (i.e., region, large basin) for reference evapotranspiration-ETo estimation, based on limited climatological and hydrological data. However, we are aware that such a method would still require further adjustment and calibration, considering the local conditions of the study site. In this context, even well-recognized methods in use so far, such as Penmen–Monteith (PM), require adjustment on many occasions [6,8]. Over the last 50 years, several scientists developed different methods to calculate the ETo; however, these methods have been subjected to local conditions and have several limitations to their applicability on a large scale. One of the most popular methods that have been validated and recommended by the Food and Agriculture Organization (FAO) is the PM method, and it is now recognized as FAO 56 PM. Although the FAO 56 PM method has been proved to be the most accurate one and achieved the most minimal errors, it required much information to be applied, which is mostly unavailable in several areas worldwide. Therefore, considering physical parameters and physically-based formulas might not be feasible and/or realistic for estimating the ETo.

Due to the complexity of estimating the ETo-based physical process, several machine learning methods were employed to estimate the ETo during the last two decades of the 21st century [8]. More recently, during the last two decades, artificial neural network (ANN) models have been examined for estimating the ETo, such as the multi-layer perceptron neural network (MLP-NN), fuzzy logic (FL), the adaptive neuro-fuzzy neural network (ANFIS), and least square support vector regression (LSSVR) [8,9,10,11]. The motivation for utilizing the ANN models is that these methods can provide high accuracy and robustness, are modeless, and can easily handle big data [12,13].

Although the ANN showed outstanding performance for estimating the ETo, there is a need to include several meteorological and hydrological variables in the model input in order to achieve relatively high estimating accuracy for the ETo utilizing ANN, [14,15]. Additionally, it has been reported in a few studies that the ETo prediction model using the ANN experienced a few challenges, such as over-fitting and the selection of the appropriate input variables based on the climate conditions of the study area [16].

Recently, the M5 model tree (M5RT) has been developed and considered as one of the most common methods used for simulation [17]. The process of the M5RT is designed to divide the data into sub-regions and consider the tree applying data of each sub-region. The input space should be divided into sub-regions, and then a fitted linear model could be used for each sub-region, [18]. There are a few studies which showed that the M5RT could achieve proper estimation accuracy but requires extensive sampling input data [19]. In general, M5RT is considered a straightforward procedure that could be worthwhile for estimation applications, especially with the availability of quite large amounts of data [17,20,21].

Furthermore, the ANFIS method has successfully shown a high ability to provide accurate predictions in different engineering applications, especially for the hydrological and climatological variables [22,23,24,25]. Even with a limited amount of available data of climate parameters, ANFIS could achieve accurate estimation for daily pan evaporation [24]. Further enhancement of the ANFIS model, including either grid partitioning (GP) or subtractive clustering (SC), has been developed by Sanikhani et al. [26]. It showed outstanding performance for estimating the daily evaporation; however, it required several input variables and a relatively large amount of data. More recent novel enhancement for the ANFIS model has been developed, which is defined as a dynamic evolving neural-fuzzy inference system (DENFIS) in order to be more suitable for dynamic time series prediction applications [27,28]. Conceptually, the DENFIS is designed to generate and update new fuzzy rules during the learning of the system. Such a procedure allows the DENFIS to calculate the desired output according to them-most activated rules, which have been chosen dynamically from the set of the fuzzy rules [27,28,29]. It could be noticed from the DENFIS structure and procedure and the nature of ETo that the DENFIS model has the potential to adequately and effectively estimate the ETo. To the knowledge of the authors, there is no study in the literature related to the application of DENFIS in modeling ETo.

More recently, LSSVR has been known as a useful tool for several engineering prediction applications. LSSVR has a simple and effective structure; however, it includes three unknown parameters that should be estimated and initialized at the beginning, which is considered one of the major disadvantages of the LSSVR. Kisi [19] showed that the more accurate the estimation of the LSSVR’s unknown parameters, the more accurate the estimation of the desired variable. However, the most common method to estimate these three unknown parameters is based on trial and error, as in many previous studies. One innovation for the development of a successful LSSVR model is to be integrated with a suitable optimization algorithm that could be able to search for the optimal estimation values for these three unknown parameters [19]. It has been reported that the gravitational search algorithm (GSA) is a useful optimization algorithm which is comparatively fast in convergence and free from trapping in local minima over the other optimization algorithms [30]. Therefore, integrating the LSSVR with (GSA) as an efficient optimizer for searching for the optimal values of the LSSVR’s unknown parameters could be a suitable solution to enhance the LSSVR’s ability to estimate the desired ETo. Available literature indicates that new methods are essential to enhancing ETo estimation preciseness and decreasing the model’s uncertainty. For this reason, novel hybrid heuristic soft computing methods were examined in this study for performing effective evapotranspiration modeling. A number of studies [31,32,33,34,35] have compared one or more of the hybrid heuristic models using several meteorological variables at different time steps, and the results obtained have shown that hybrid heuristic soft computing methods generally provided good accuracy. However, even though the aforementioned studies demonstrated successful applications of hybrid heuristic soft computing models, the most important point to note is that, generally speaking, these models do not use fewer independent input variables; instead, they require several inputs—i.e., U, RH, SH, Tmean, Tmax, and Tmin—and among them the U, RH, SH, and SR are the meteorological variables reported as the most significant factors influencing ETo and of primary importance. To the author’s knowledge, there is not any published study that investigates the potential of the LSSVR-GSA method in modeling ETo. First, we developed the hybrid of GSA and LSSVR for modeling ETo. Second, we used the already applied standalone soft computing method (M5RT) with only average temperature input, whereas most of the other studies in the literature used a mix of the Tmax and Tmin and several other meteorological variables during applications of such models. Third, we used extraterrestrial radiation which is easily obtained from the Julian date and latitude information as input to the models. Fourth, we demonstrated the effect of periodicity (month number) as an input variable for ETo estimation using limited climatic inputs.

In the current study, an attempt to develop a model to estimate the ETo based on a minimal number of input variables has been proposed utilizing advanced machine learning methods. LSSVR-GSA, DENFIS, and M5RT models have been developed to estimate the ETo considering only the temperature as an input variable. Data from three stations in the Jinsha river basin of China have been used in order to evaluate and examine all the proposed models. Additionally, in-depth, comprehensive analyses for the performance of all models have been accomplished in order to elucidate the logic and the cause behind each model’s estimation accuracy.

## 2. Materials and Methods

### 2.1. Case Study

**Figure 1:**
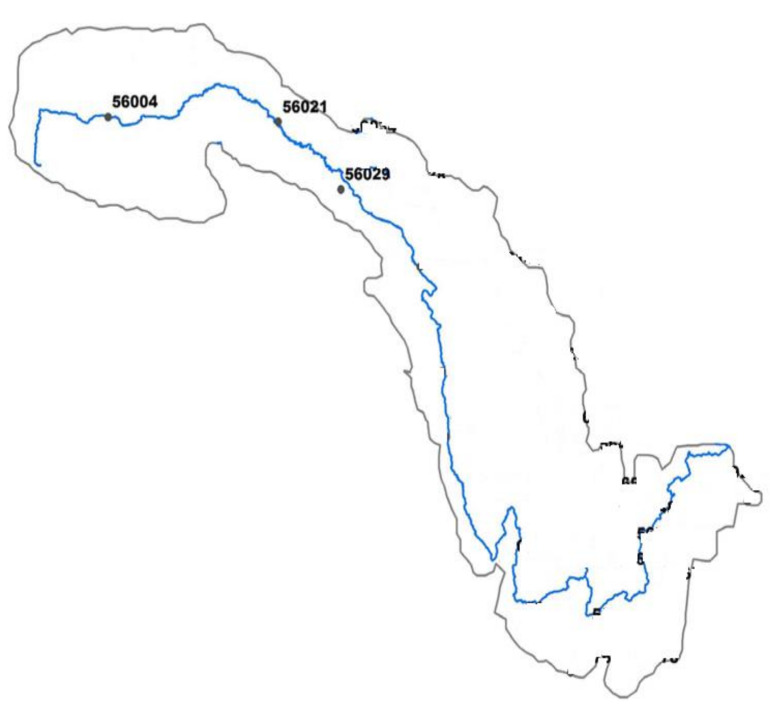
Study area.

The study uses monthly average temperatures (T), extraterrestrial radiation (Ra), and evapotranspiration (ETo) data of three meteorological stations, represented as 56004 (latitude 34.21 N, longitude 92.43 E), 56021 (latitude 34.13 N, longitude 95.78 E), and 56029 (latitude 33.01 N, longitude 97.01 E,) stations situated in the Jinsha river basin of China. The location of each station can be seen in Figure 1. These stations are operated by the China Meteorological Administration (CMA). Jinsha river basin consists of three provinces (Qinghai, Yunan, Sichuan, and Tibet Autonomous) covering the 473.2 × 10^3^ km^2^ drainage area of China. The Jinsha river basin has a very vital role in regional and national economic development due to its contribution to irrigation, water supply, flood control, wood drift, tourism, and plentiful hydropower resources (58,060 MW). The mean annual rainfall in the Jinsha river basin is 750 mm, 90% of which occurs from May to October. The basin selected area belongs to humid warm temperate, which is the primary source of water, having annual mean rainfall of about 1200 mm with mean annual potential evapotranspiration of about 1350 mm.

In the study, the data periods of three meteorological stations from 1961 to 2012 were used. Available data were divided into three parts as training (50% of the whole data covering 1961–1986), validation (25% of the whole data covering 1986–1999), and testing (25% remaining part covering 2000–2012). The brief statistical characteristics of the user data are summed up in Table S1. Evapotranspiration data of three meteorological stations are calculated using the FAO-56 PM method. The detailed procedure of the FAO-56 PM method is described by Allen et al. [36] and She et al. [37].

### 2.2. Methods

#### 2.2.1. Dynamic Evolving Neural-Fuzzy Inference System (DENFIS)

DENFIS is a neural-fuzzy modeling approach that evolves through gradual hybrid learning procedures resulting from the tuning of local elements regarding the new data entry to the model-specific categorization [27]. DENFIS is an extension of the evolving fuzzy neural network (EFuNN), which uses a clustering approach called the evolving clustering method (ECM) [38]. Dynamic features of the EFuNN make DENFIS useful for online adaptive systems also. ECM itself is an online fast-evolving clustering method that considers the maximum distance between points and the cluster’s center.

ECM is based on triangular membership functions, which serve as fuzzy sets and the utilization of an alternative weighting scheme for local learning of resulting parameters. Usually, the input and output neurons can be fuzzified by a fuzzy quantization approach [39]. The offspring fuzzy rules are consequently generated and updated while the system is in operation. The outputs resulting from DENFIS are computed through FIS-based activated fuzzy rules, which are dynamically chosen from a given fuzzy set of rules. Notably, the ECM used in the DENFIS interface utilizes a scatter partitioning of the input space to create fuzzy inference rules [38]. Uses of ECM improve the computational performance for the dynamic estimation of the cluster’s quantity in the given dataset by facilitating the finding of their current centers in the input space. A typical structure and detailed description of the EFuNN interface based on ECM from which the DENFIS approach is derived can be found at [27]. DENFIS uses a typical model architecture, which is based on the Takagi–Sugeno type fuzzy inference engine [34]. In this regard, DENFIS utilizes a model-based approach called the “lazy” learning approach. According to this approach, the fuzzy network estimates the position of each input vector in the attribute space and forms accordingly; meanwhile, a fuzzy inference system which predicts the output through a dynamic process is created during the incremental learning.

The classical “lazy” learning process of the fuzzy network utilizes a sample-based approach, wherein a small local model on demand is constructed regarding local samples taken from the closest query point. The learning process and governing equations used in the DENFIS model and applied in this study are described in detail in [39]. DENFIS models are being applied in different water resource-related areas and other disciplines as well. The use of DENFIS in rainfall-runoff modeling showed that results attained from the local learning model were significantly better than results achieved from physically-based models [38]. In another study related to evaporation modeling based on limited meteorological data, it was found that DENFIS models increased the accuracy of the outputs significantly [39]. Similar results were obtained in other studies as well [40]. DENFIS models were recently applied to predict solar radiation [41]; the results showed that DENFIS provides faster and much more accurate results compared to the other neuro-fuzzy models.

#### 2.2.2. Least Squares Support Vector Regression (LSSVR)

Least squares support vector regression was initially proposed by [42]; it is a version of the support vector regression (SVR) algorithm improved by adopting the loss function differently from SVR and minimizing the square error. In the LSSVR interface model, the inequality constraints have been replaced with equality constraints by transforming quadratic programming issues into a linear equation to overcome the calculation issue of the large-scale dataset [43]. SVR itself is well known, and a robust algorithm applied in different areas mainly for data regression and also to overcome the over-fitting issue. LSSVR differs from SVR, mainly due to the types of functions used by each of them. LSSVR handles square errors instead of non-negative errors, and it uses equality constraints instead of inequality constraints, as opposed to the conventional SVR [44].

**Figure 2:**
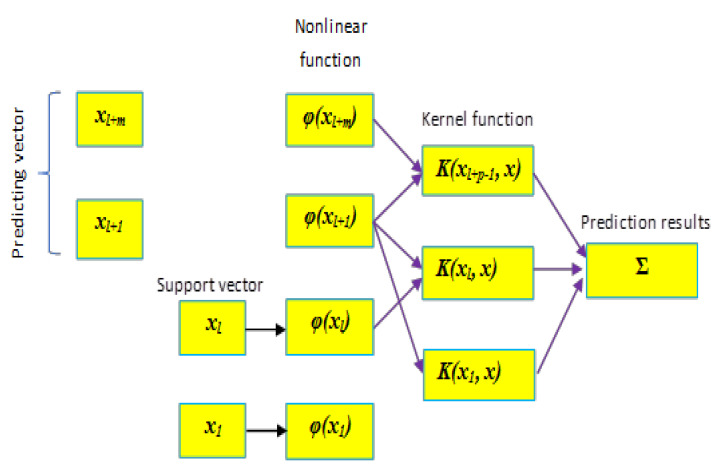
LSSVR model for evapotranspiration (ETo) modeling.

Similarly, to the DENFIS, LSSVR performs the training procedure in terms of solving a linear system instead of using a quadratic programming problem, which thus significantly improves the computation time and the accuracy of the model learning ability [45]. Figure 2 illustrates the structure of a typical LSSVR model. A detailed explanation about LSSVR applications, parameters, and respective governing equations is given by Yuan et.al [44]. LSSVR is widely applied in solving challenging problems in different areas. Wu and Peng [43] used LSSVR to improve the prediction performance of the potential wind power; they found that LSSVR can enhance the accuracy of up to 20% compared to other single or hybrid models. A similar conclusion was obtained by Lu et al. [46] who applied LSSVR for short-term forecasting of wind power. The application of the LSSVR has shown promising results in the prediction of nanoparticles as well [47].

#### 2.2.3. Gravitational Search Algorithm (GSA)

GSA, which is based on the law of gravity and motion proposed initially by Rashedi et al. [30], represents one of the most effective optimization algorithms compared to other evolutionary algorithms. In GSA, each nod is characterized by four parameters: position, inertial mass, gravitational mass, and velocity. The location of each node corresponds to a solution of the problem, while the gravitational and inertia masses for each node were obtained utilizing a fitness function [48]. The location of the nodes can be expressed as follows:

(1) $$X_{i}=(x_{i}^{1},\ldots ,x_{i}^{k},\ldots ,x_{i}^{s})\;i=1,2,\ldots ,\mathrm{np}$$

where $x_{i}^{k}$ represents the location of the ith node for the kth dimension. The mass of each node is computed after calculating the fitness of the given population as follows:

(2) $$X_{i}=\frac{fit_{i}\left(t\right)-worst\;\left(t\right)}{best\left(t\right)-worst\;\left(t\right)}$$

(3) $$M_{i}\left(t\right)=\frac{m_{i}\left(t\right)}{\sum_{j=1}^{N}m_{j}\left(t\right)}$$

where *fit*_*i*_(*t*) and *M*_*i*_(*t*) represent the fitness value and mass of the *i*th node at time *t*, respectively, whereas best(*t*) and worst(*t*) are the minimum fitness value and maximum fitness value, respectively, the gravitational acceleration of the node *i*, is calculated as follows: firstly, the force exerted by a large node on the node *i* is computed.

(4) $$F_{ij}^{k}\left(t\right)=G_{c}\left(t\right)\frac{M_{i}\left(t\right).M_{j}\left(t\right)}{||x_{i}\left(t\right),x_{j}{\left(t\right)||}_{2}+\varepsilon }.\;\left(x_{i}^{k}\left(t\right)-x_{j}^{k}\left(t\right)\right)$$

where *M*_*i*_(*t*) and *M*_*j*_(*t*) are the passive and active gravitational mass, respectively, corresponding to nodes *i* and *j* at the *t* generation; *Gc*(*t*) and *ε* are gravitational and small constants; *xk* *i* (*t*) and *xk* *j* (*t*) indicate the positions of the kth dimensions of nodes *i* and *j* at the *t* generation; and ‖*xi*(*t*), *xj*(*t*)‖ is the Euclidean distance between nodes *i* and *j*. The total gravitational acceleration of the *i*th node was calculated using the law of motion as follows:

(5) $$a_{i}^{k}\left(t\right)=\frac{\sum_{j=1,\;j\neq i}^{N}rand.\;F_{ij}^{k}\left(t\right)}{M_{i}\left(t\right)}$$

where $a_{i}^{k}\left(t\right)\;$ represents the gravitational acceleration of the node *i* in the kth dimension, and the rand represents a random variable with uniform distribution within the interval (0,1). The total gravitational force exerted on the node *i* in the kth dimension is calculated as follow:

(6) $$F_{ij}^{k}\left(t\right)=M_{i}\left(t\right)xa_{i}^{k}\left(t\right)=\overset{N}{\underset{j=1,\;j\neq i}{\sum }}rand.\;F_{ij}^{k}\left(t\right)$$

Afterward, the speed and location of each node were updated as follows:

(7) $$v_{i}^{k}\left(t+1\right)=rand\;v_{i}^{k}\left(t\right)+a_{i}^{k}\left(t\right)$$

(8) $$x_{i}^{k}\left(t+1\right)=x_{i}^{k}\left(t\right)+v_{i}^{k}\left(t+1\right)$$

As highlighted above also, GSA utilizes the gravitational force as the direct form with which to communicate the cooperation of the nodes. The heavy nodes in GSA are processed, infer reasonable solutions, and move more gradually than lighter ones. Thus, the GSA searches for the ideal solution by appropriately calibrating the inertia and gravitational masses of nodes where every node provides a specific solution.

#### 2.2.4. HLGSA (Hybrid LSSVR-GSA)

The combination of LSSVR with the gravitational search algorithm (GSA) has been reported to improve the forecasting accuracy and computational performance [43] significantly. To our knowledge, there are only a few studies applied to hybrid models, such as combining GSA with other ANN models. Yuan et al. [44] applied LSSVM–GSA to predict day-ahead wind power; they concluded that GSA provided higher accuracy and improved the computational duration. Similar conclusions were reported by Lu at al. [46] and Sun at al. [47] in the case of PM_2.5_ concentration prediction. The process of the evapotranspiration prediction model HLGSA using the hybrid of LSSVR and GSA methods consists of the following steps:

1. Firstly, divide meteorological datasets into training, validation, and testing parts.
2. Second, select the RBF kernel function and initial parameters for the HLGSA method to build the initial LSSVR model. The initial values of the parameters are set as follows: the range of penalty factor *γ* is 0.1 to 1500, the range of RBF parameter *σ*^2^ is 0.001 to 10, the iteration range is 20–30, the number of particles can be set up to 30, and constant alpha was found to perform better in range of 12–18, whereas initial gravitational constant *G*_0_ was found to perform better in the range from 102 to 120.
3. Third, compute the particle fitness value of each node. The RMSE is selected as the fitness function in this study. The fitness function of this method is defined as follows:
4. Fourth, chose the best parameter combination through GSA to obtain the optimal values for the LSSVR parameters.
5. Fifth, utilize the new combination of parameters to reconstruct the LSSVR if it does not meet the stopping criterion.
6. Last, the optimal LSSVR model for forecasting evapotranspiration was built based on the typical parameter values.

#### 2.2.5. M5 Model Tree

M5 model tree (M5RT) is a novel neural approach developed initially by Quinlan [49] to help in solving continuous class learning problems. This model is based on a binary tree that serves as the backbone for the model itself. An M5 model tree consists of a linear regression function applied to terminal leaf nodes to describe the relationships of independent and dependent variables. Since the M5 model tree is a quantitative data focus model, this feature increases the accuracy, and as a result, the importance compared to other models [50]. In general, the principle of the tree-based model consists of “divide-and-conquer” approach for constructing a relationship between independent and dependent variables or input and output parameters. They can also be used for qualitative and quantitative data assessment [51]. All model trees can effectively learn and succeed in tasks with very high dimensionality. The main advantage of the M5 model tree compared to regression trees and other models is that it is much smaller than regression trees [52]. Additionally, the decision strength is evident, and the regression functions do not usually contain abundant variables, which in some cases can reduce the computational performance.

**Figure 3:**
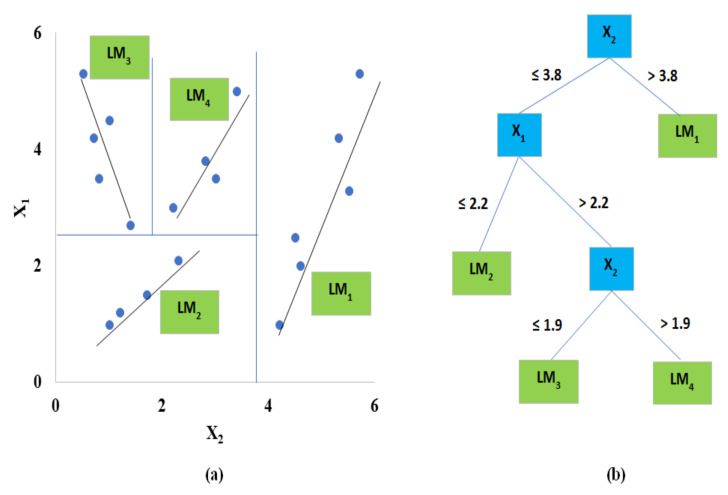
Schematic view of M5 model tree (**a**) structure and (**b**) splitting data space into sub-regions.

M5 model tree consists of two steps. The first step has to do with the splitting of the datasets into subsets. Splitting process: it often creates an excessive tree-like structure that leads to overfitting. In the second step, the overrun tree is trimmed; then trimmed subtrees are replaced with linear regression functions (Figure 3). M5 model tree has found many applications in water resource-related areas. Rahimikhoob et al. [53] applied the M5 model tree to compute evapotranspiration (ETo) in a semi-arid region. They found that the M5 model tree provides more robust results compared to other conventional or empirical methods. However, for the arid zone, Rahimikhoob [54] found that ANN estimated ET0 better than the M5 model tree.

Nevertheless, both models performed in agreement for that study case, and the results were very similar to the results obtained from the FAO56-PM method. Kisi and Kilic [52] estimated ETo considering several empirical and neural-fuzzy approaches, including the M5 model tree. They found that the M5 model tree showed better accuracy, particularly comparing to the empirical models.

## 3. Application and Results

The potential of new machine learning methods, LSSVR-GSA and DENFIS, was investigated by applying them to temperature (T) and extraterrestrial radiation (Ra) data of three stations located in China, and the results were compared with the well-known M5RT method. The models’ accuracy was evaluated based on three statistical criteria: root mean square error (RMSE), mean absolute error (MAE), and determination coefficient (R^2^). The RMSE and MAE can be defined as:

(9) $$\mathrm{MAE}=\frac{\;\;\sum_{i=1}^{N}\left|ET_{im}-ET_{ip}\right|}{N}$$

(10) $$\mathrm{RMSE}=\sqrt{\frac{\sum_{i=1}^{N}{\left(ET_{im}-ET_{ip}\right)}^{2}}{N}}$$

where *N* is the number of datapoints used, $ET_{\mathrm{ip}}$ is predicted ETo, and $ET_{\mathrm{im}}$ is ETo calculated by FAO 56 PM.

Table S2 sums up the validation and test results of the and M5RT models concerning different input combinations comprising previous values of temperature (T) and extraterrestrial radiation (Ra) for the first station (56004). In Table S2, T_t_ and Ra_t_ refer to the mean air temperature and extraterrestrial radiation at time t and vice versa. In the tables, t represents the month which ETo needs to be predicted, whereas t-1 represents the previous month.

It is apparent from Table S2 that the LSSVR-GSA has slightly better accuracy than the DENFIS, and they both perform superiorly to the M5RT in modeling monthly ETo. There are not large differences between the optimal T-based and Ra-based models; the RMSE differences are 12.6%, 12.9%, and 5.9% for the LSSVR-GSA, DENFIS, and M5RT, respectively. It is worth noting that Ra-based models only use Julian date and do not require temperature measurement.

In Table 1, the model results are presented for the optimal (best) inputs, including periodicity input (α), which indicates the month number of the output. In the table, Opt T and Opt Ra refer the optimum T and optimum Ra inputs, which provided the best accuracy in the test stage (see Table S2); Opt T based models for LSSVR-GSA, DENFIS, and M5RT are found as T_t_, T_t-1,_ T_t-2_, T_t_, T_t-1__,_ T_t-__2,_ T_t-__3_, T_t_, T_t-1__,_ and T_t-__2_ respectively. For the optimal Ra input combination, Ra_t_, Ra_t-1__,_ Ra_t-__2,_ and Ra_t-__3_ input combination provided the best results for DENFIS and LSSVR-GSA models, whereas, Ra_t_, Ra_t-1__,_ Ra_t-__2_ provided best results for the M5RT model. As clearly observed from Table 1, the models’ performances are also examined by considering both optimum temperature and extraterrestrial radiation inputs. As evident from the table, including periodicity input marginally improves the models’ accuracy, and combining optimal T and optimal Ra inputs worsens the performances of the three methods in prediction of monthly ETo.

**Figure 4:**
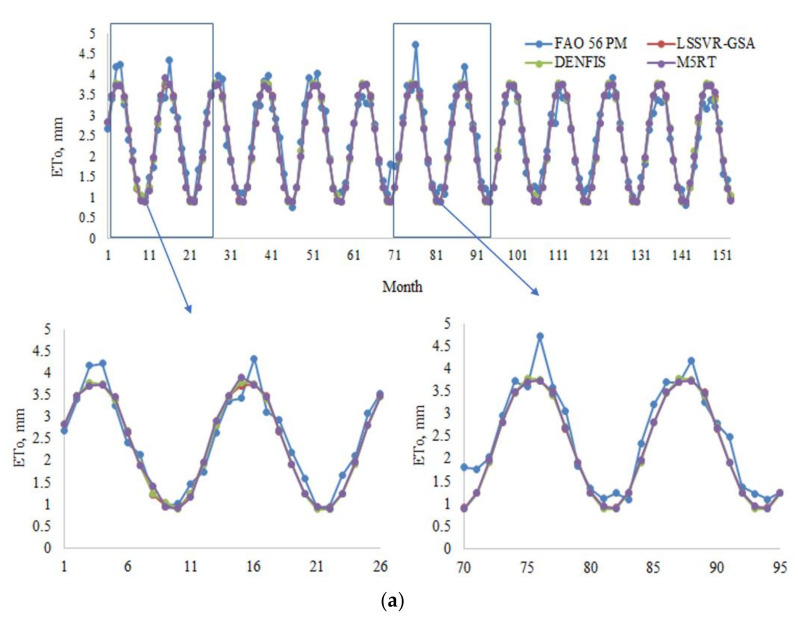

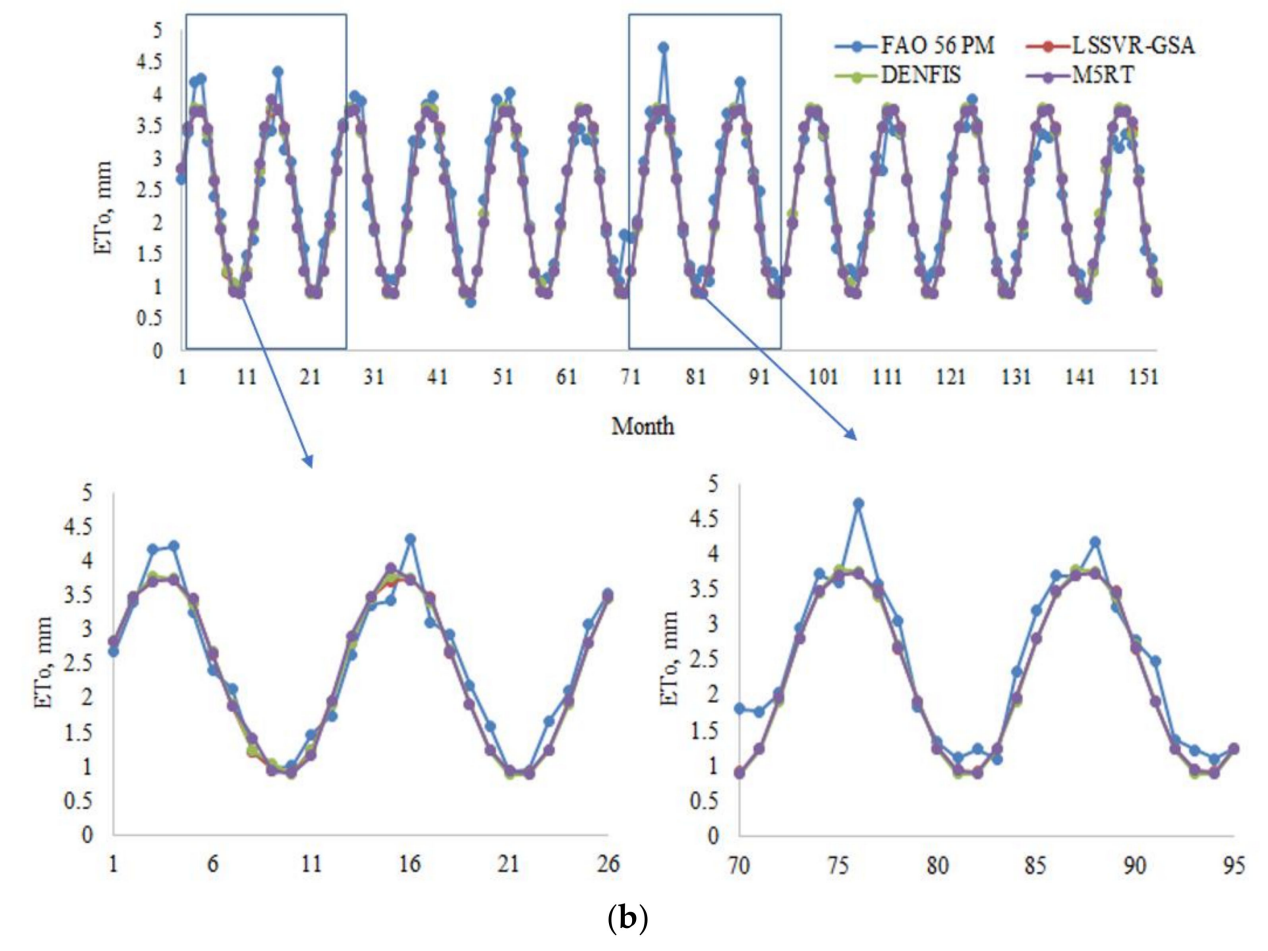
(**a**) Time variation graphs of the FAO 56 PM and estimated ETo by LSSVR-GSA, DENFIS, and M5RT in the test period of Station 56004 using optimal T inputs. (**b**) Time variation graphs of the FAO 56 PM and estimated ETo by LSSVR-GSA, DENFIS, and M5RT in the test period of Station 56004 using optimal Ra inputs.

**Figure 5:**
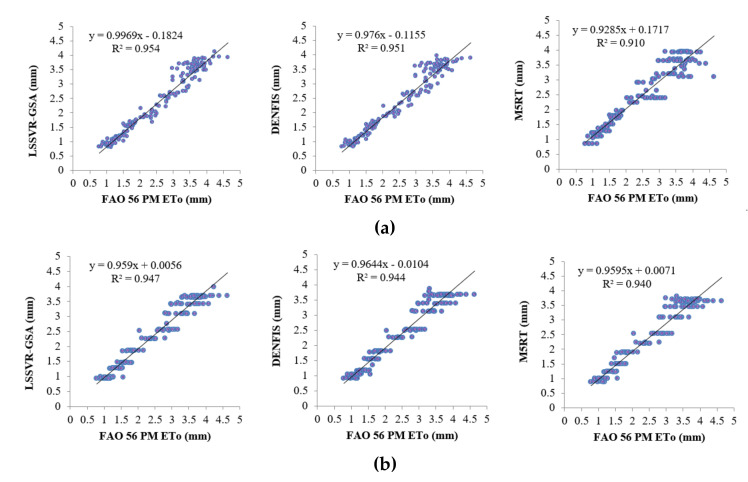
Time scatterplots of the observed and estimated ETo (by LSSVR-GSA, DENFIS, and M5RT) in the test period of Station 56029 using optimal (**a**) T and (**b**) Ra inputs.

Model estimates of each method with Opt T and Opt Ra inputs in the test period are compared in Figure 4a,b in the form of hydrograph for the first station (56021). It can be seen from the zoomed sections that the LSSVR-GSA is closer to the FAO 56 PM ETo than the DENFIS and M5RT models. Figure 5 makes a scatterplot comparison of the model estimates. As seen from the graphs, LSSVR-GSA has less scattered points, closely followed by the DENFIS model, for both temperature and extraterrestrial radiation inputs.

Validation and test statistics of the three applied methods are reported in Table S3 for the prediction of monthly ETo of the second station (56021). It is observable from Table S3 that the temperature-based LSSVR-GSA models outperform the corresponding DENFIS and M5RT models. The best LSSVR-GSA model has lower RMSE (0.230 mm) and MAE (0.179 mm) and higher R^2^ (0.956) compared to the other two best models. In the case of extraterrestrial radiation input, however, LSSVR-GSA has slightly better accuracy than the DENFIS and M5RT models. It is worth noting here that Ra-based models perform superiorly regarding the T based models, except LSSVR-GSA, so that there is a marginal difference between R-based and T-based models. This result caries practical importance because R-based models only use Julian’s date, and they can predict monthly ETo without climatic data. For station 56021, the optimal T inputs were found T_t_, T_t-1,_ and T_t-2,_ for LSSVR-GSA and M5RT models, whereas they were T_t_, T_t-1,_ T_t-2,_ and T_t-3_ for DENFIS model.

The optimal models with the periodicity information are compared in Table 2 for the second station (56021). In this station, also adding periodicity information slightly improves prediction accuracy, and in some cases, it negatively affects the models’ accuracies (e.g., see DENFIS with Opt Ra input and M5RT with Opt T and Opt T, Opt Ra inputs). Here, also combining optimal temperature and extraterrestrial radiation inputs does not improve the models’ exactness compared to optimal T and/or optimal Ra input-based models.

**Figure 6:**
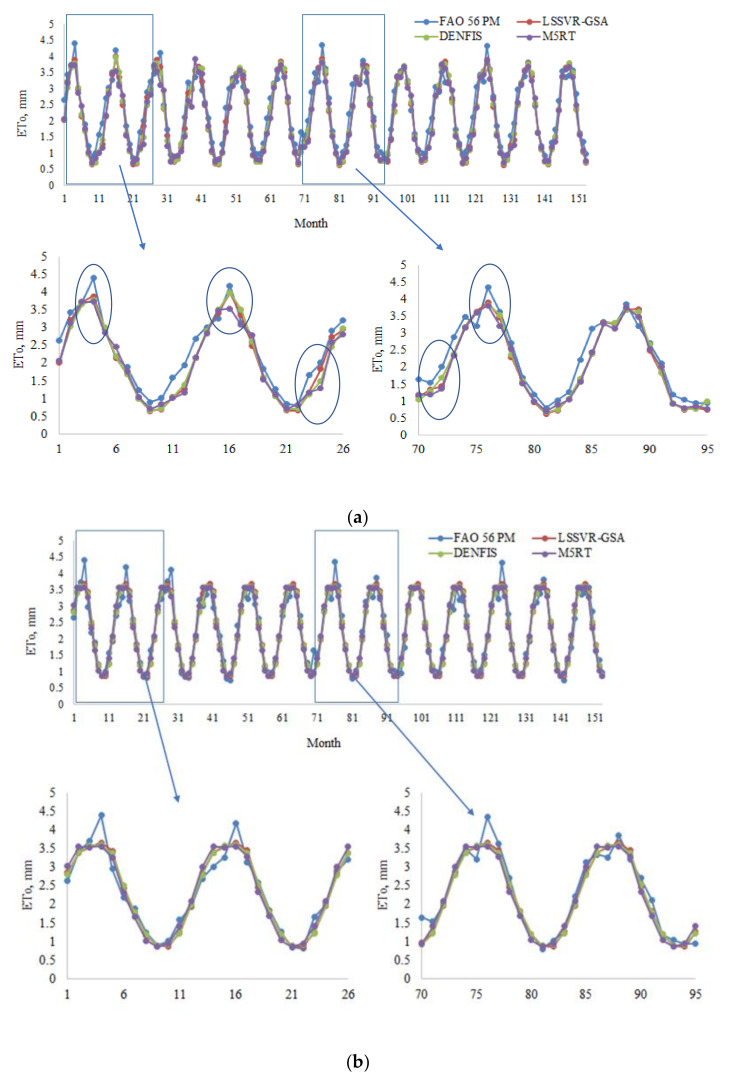
(**a**) Time variation graphs of the FAO 56 PM and estimated ETo (by LSSVR-GSA, DENFIS, and M5RT) in the test period of Station 56021 using optimal T inputs. (**b**) Time variation graphs of the FAO 56 PM and estimated ETo by LSSVR-GSA, DENFIS, and M5RT in the test period of Station 56021 using optimal Ra inputs.

**Figure 7:**
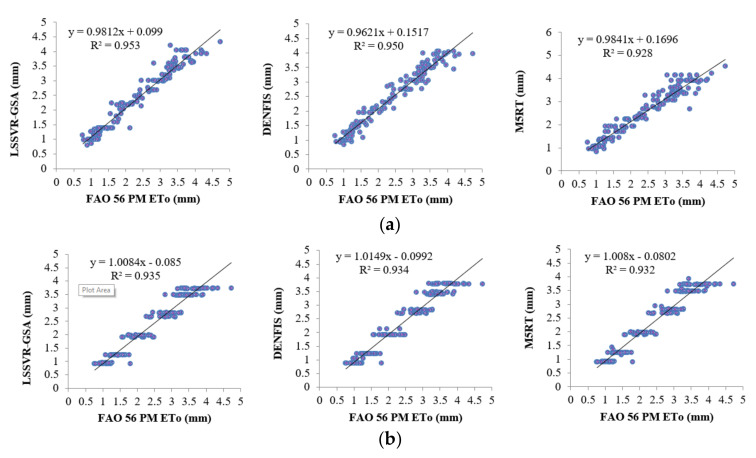
Time scatterplots of the observed and estimated ETo (by LSSVR-GSA, DENFIS, and M5RT) in the test period of Station 56021 using optimal (**a**) T and (**b**) Ra inputs.

Figure 6a,b illustrate the ETo estimates of three methods with Opt T and Opt Ra inputs in the test period for the second station (56021). As can be seen, the LSSVR-GSA estimates are closer to the FAO 56 PM ETo than the DENFIS and M5TR models. It can be said that the fluctuations of the model estimates are very close to each other in the case of Opt Ra input (Figure 6b). This confirms the statistics provided in Table 2. Scatterplot comparison of the model estimates is shown in Figure 7. It is evident from the graphs that the LSSVR-GSA has less scattered estimates compared to the other two models and is closely followed by the DENFIS. In the case of Ra input, the distributions of all three models are similar to each other.

Table S4 gives the validation and test statistics of the LSSVR-GSA, DENFIS, and M5RT models for prediction of monthly ETo of the third station (56029) using previous values of temperature and extraterrestrial radiation to find their optimal input combinations. In this station, the LSSVR-GSA method performs better than the DENFIS and M5RT methods; however, there is a slight difference among the Ra-based methods, similarly to the previous station (56021). Among the all models, the LSSVR-GSA with T_t_, T_t-1_, T_t-2_, and T_t-3_ inputs has the lowest RMSE (0.230 mm) and MAE (0.172 mm) and the highest R2 (0.954) in monthly ETo prediction.

Table 3 compares the optimal T- and R-based models, together with periodicity input (α). For the LSSVR-GSA method including α generally, it worsens the prediction accuracy, while the performances of DENFIS models are considerably improved by importing periodicity input; the most considerable improvement is 25% for the model with Opt T input. Combining optimum T and optimum Ra inputs also considerably improve the prediction accuracy of DENFIS; improvements are 22% and 10% compared to Opt T and Opt Ra inputs. For this station, it can be concluded that the DENFIS model with Opt T, α input performs better than the other models.

**Figure 8:**
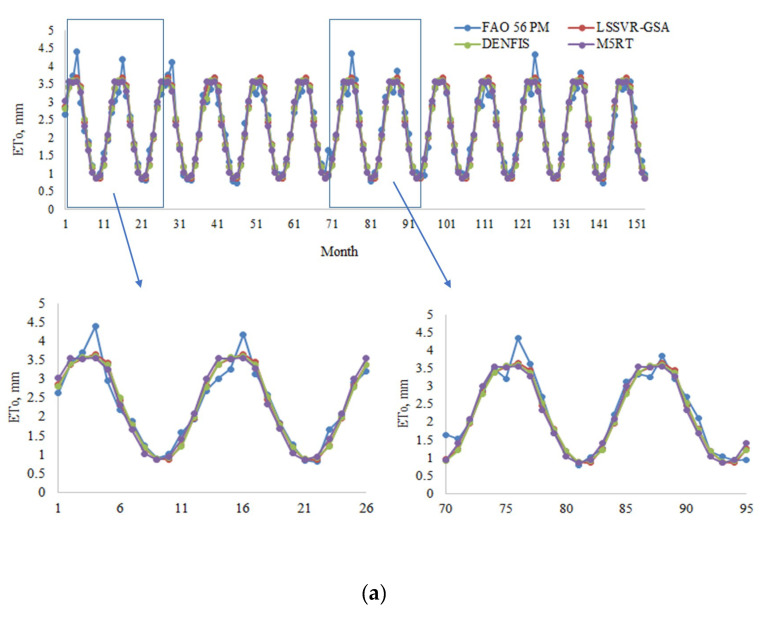

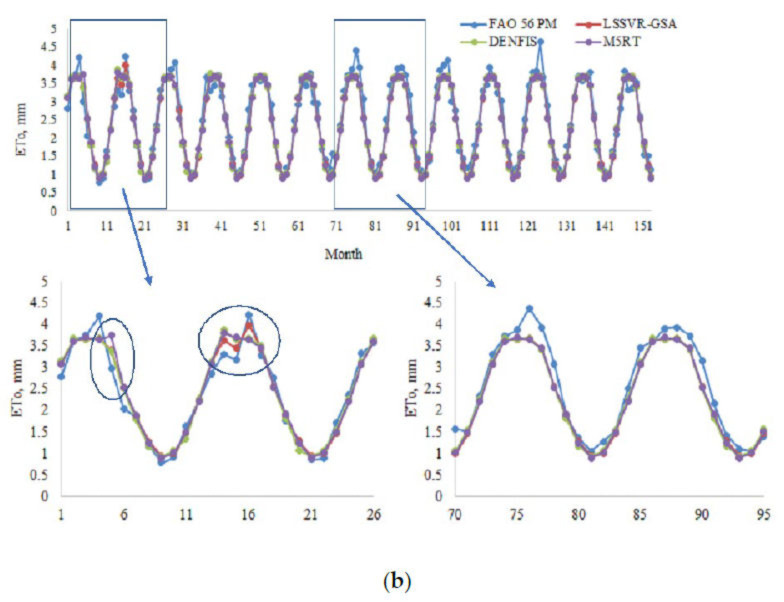
(**a**)Time variation graphs of the FAO 56 PM and estimated ETo (by LSSVR-GSA, DENFIS, and M5RT) in the test period of Station 56029 using optimal T inputs. (**b**) Time variation graphs of the FAO 56 PM and estimated ETo by LSSVR-GSA, DENFIS, and M5RT in the test period of Station 56029 using optimal Ra inputs.

**Figure 9:**
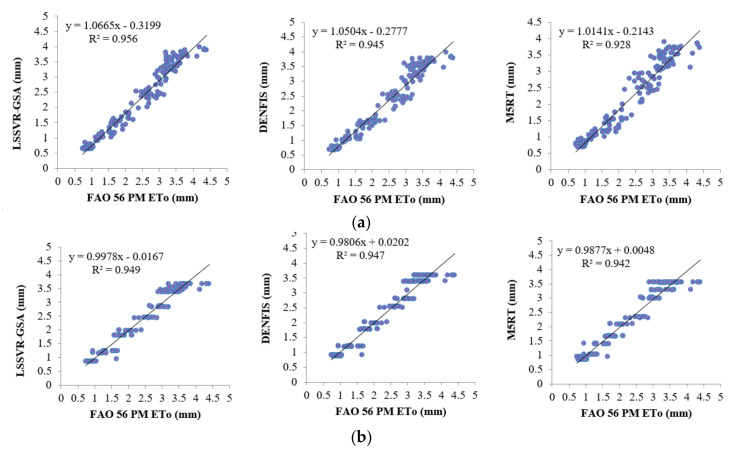
Time scatterplots of the observed and estimated ETo (by LSSVR-GSA, DENFIS, and M5RT) in the test period of Station 56021 using optimal (**a**) T and (**b**) Ra inputs.

Figure 8a,b provide the ETo estimates of three methods with Opt T and Opt Ra inputs in the test period for the third station (56029). In this station, the difference between LSSVR and the other two methods is more clearly observed, mainly for the optimum temperature inputs. Figure 9 compares the models’ estimates concerning their scatterplot distributions. The LSSVR-GSA has the estimates which are closer to the FAO 56 PM ETo with less scattered distribution compared to DENFIS and M5RT models. In the case of Ra input, the other two models and are closely followed by the DENFIS. In the case of Ra input, however, the distributions of all three models are very close to each other.

## 4. Conclusions

The potentials of two new machine learning methods, least-square support vector regression with a gravitational search algorithm and the dynamic evolving neural-fuzzy inference system, for modeling reference evapotranspiration using only temperature data, were investigated in the study, and estimates of the new methods were compared with the well-known M5 model tree method. Previous values of air temperature and extraterrestrial radiation were tried as inputs to the models to estimate monthly ETo. Three stations from China were used as case studies. The results obtained from the applications provided the following conclusions:

1. In all three stations, the temperature or extra-terrestrial radiation-based LSSVR-GSA models performed superiorly to the DENFIS and M5RT models in estimating monthly ETo. In some cases, especially for the Ra based models, however, a slight difference was found between the LSSVR-GSA and DENFIS methods, while the M5RT provided the worst estimates.
2. The results revealed that the only extra-terrestrial radiation input might provide better prediction accuracy for all the three methods, and this implies that the monthly ETo can be easily calculated with only Julian date or without temperature information.
3. Importing periodicity information to the model’s inputs generally improved the prediction accuracy, and the accuracy of DENFIS was considerably increased in the third station (56029).
4. Combining optimum air temperature and extra-terrestrial radiation inputs generally did not increase the accuracy of the methods in terms of estimation of monthly ETo. In one station (third station), however, the combination of both inputs improved the accuracy of DENFIS methods by 22% and 10% (RMSE) compared to optimum T and optimum Ra inputs, respectively. In this station, this method performed better than the LSSVR-GSA and M5RT.

The results of this study recommend the use of the LSSVR-GSA model as an efficient tool for estimating monthly ETo using only temperature or extraterrestrial radiation. This is very important in practical applications, especially for the developing countries, where some climatic data may be missing or absent because of technical reasons or lack of opportunities.

## Figures and Tables

**Table 1:** Validation and test statistics of the models in monthly ETo prediction using the optimal inputs of T, Ra, and α—Station 1 (56004).

Model Inputs	Validation Period			Test Period		
	RMSE	MAE	R^2^	RMSE	MAE	R^2^
LSSVM-GSA						
Opt T	0.246	0.184	0.958	0.246	0.182	0.953
Opt T, α	0.243	0.180	0.959	0.244	0.180	0.955
Opt Ra	0.341	0.246	0.920	0.277	0.219	0.935
Opt Ra, α	0.339	0.244	0.922	0.274	0.217	0.936
Opt T, Opt Ra	0.285	0.210	0.940	0.301	0.226	0.930
Opt T, Opt Ra, α	0.284	0.209	0.942	0.299	0.225	0.932
DENFIS						
Opt T	0.271	0.198	0.947	0.249	0.190	0.950
Opt T, α	0.263	0.193	0.950	0.246	0.185	0.952
Opt Ra	0.342	0.248	0.919	0.281	0.221	0.934
Opt Ra, α	0.341	0.250	0.920	0.280	0.221	0.935
Opt T, Opt Ra	0.288	0.212	0.939	0.305	0.229	0.927
Opt T, Opt Ra, α	0.286	0.211	0.940	0.304	0.228	0.928
M5RT						
Opt T	0.322	0.221	0.923	0.305	0.232	0.928
Opt T, α	0.304	0.215	0.931	0.323	0.239	0.931
Opt Ra	0.350	0.266	0.917	0.323	0.267	0.932
Opt Ra, α	0.348	0.264	0.918	0.322	0.264	0.933
Opt T, Opt Ra	0.304	0.219	0.931	0.309	0.231	0.923
Opt T, Opt Ra, α	0.301	0.215	0.933	0.306	0.230	0.925

**Table 2:** Validation and test statistics of the models in monthly ETo prediction using the optimal inputs of T, Ra, and α—Station 2 (56021).

Model Inputs	Validation Period			Test Period		
	RMSE	MAE	R^2^	RMSE	MAE	R^2^
LSSVM-GSA						
Opt T	0.235	0.161	0.954	0.230	0.179	0.956
Opt T, α	0.233	0.158	0.955	0.228	0.176	0.961
Opt Ra	0.276	0.195	0.933	0.236	0.176	0.949
Opt Ra, α	0.273	0.192	0.935	0.234	0.175	0.950
Opt T, Opt Ra	0.245	0.170	0.944	0.238	0.195	0.940
Opt T, Opt Ra, α	0.236	0.164	0.950	0.232	0.182	0.952
DENFIS						
Opt T	0.242	0.162	0.951	0.301	0.253	0.945
Opt T, α	0.240	0.162	0.952	0.227	0.171	0.961
Opt Ra	0.286	0.209	0.932	0.241	0.186	0.947
Opt Ra, α	0.288	0.213	0.930	0.268	0.207	0.942
Opt T, Opt Ra	0.276	0.187	0.944	0.284	0.215	0.938
Opt T, Opt Ra, α	0.260	0.178	0.945	0.269	0.208	0.940
M5RT						
Opt T	0.265	0.189	0.940	0.310	0.227	0.921
Opt T, α	0.303	0.220	0.929	0.341	0.260	0.908
Opt Ra	0.289	0.211	0.929	0.251	0.191	0.942
Opt Ra, α	0.281	0.205	0.931	0.250	0.185	0.943
Opt T, Opt Ra	0.300	0.216	0.930	0.336	0.258	0.905
Opt T, Opt Ra, α	0.306	0.220	0.925	0.339	0.261	0.901

**Table 3:** Validation and test statistics of the models in monthly ETo prediction using the optimal inputs of T, Ra, and α—Station 3 (56029).

Model Inputs	Validation Period			Test Period		
	RMSE	MAE	R^2^	RMSE	MAE	R^2^
LSSVM-GSA						
Opt T	0.202	0.160	0.968	0.230	0.172	0.954
Opt T, α	0.199	0.157	0.970	0.291	0.240	0.957
Opt Ra	0.230	0.179	0.957	0.262	0.205	0.947
Opt Ra, α	0.228	0.176	0.958	0.260	0.202	0.949
Opt T, Opt Ra	0.208	0.161	0.966	0.298	0.243	0.959
Opt T, Opt Ra, α	0.208	0.161	0.966	0.301	0.246	0.959
DENFIS						
Opt T	0.217	0.160	0.964	0.291	0.239	0.951
Opt T, α	0.222	0.166	0.962	0.218	0.165	0.959
Opt Ra	0.231	0.178	0.957	0.268	0.209	0.944
Opt Ra, α	0.242	0.190	0.957	0.252	0.192	0.947
Opt T, Opt Ra	0.226	0.169	0.960	0.226	0.167	0.950
Opt T, Opt Ra, α	0.221	0.166	0.961	0.219	0.158	0.952
M5RT						
Opt T	0.276	0.209	0.938	0.317	0.224	0.910
Opt T, α	0.270	0.205	0.942	0.315	0.220	0.912
Opt Ra	0.232	0.193	0.954	0.272	0.215	0.940
Opt Ra, α	0.228	0.180	0.956	0.269	0.210	0.943
Opt T, Opt Ra	0.263	0.195	0.944	0.302	0.213	0.920
Opt T, Opt Ra, α	0.260	0.193	0.946	0.299	0.210	0.922

## Supplementary Materials

The following are available online at https://www.mdpi.com/1099-4300/22/5/547/s1. Table S1: The statistical parameters of the applied data. Table S2: Validation and test statistics of the models for monthly ET0 prediction—Station 1 (56004). Table S3: Validation and test statistics of the models for monthly ET0 prediction—Station 2 (56021). Table S4: Validation and test statistics of the models for monthly ET0 prediction—Station 3 (56029).
