# Supplementary material for: Reference Evapotranspiration Modeling Using New Heuristic Methods
Source: Entropy (Basel). 2020 May 13;22(5):547. doi: 10.3390/e22050547 (PMC7517042; doi:10.3390/e22050547)
Supplement: Supplementary file 1 [file entropy-22-00547-s001.pdf]

Table S1. The statistical parameters of the applied data.

	**Station 1**				**Station 2**				**Station 3**			
	**Whole Data**	**Training**	**Validation**	**Testing**	**Whole Data**	**Training**	**Validation**	**Testing**	**Whole Data**	**Training**	**Validation**	**Testing**
						Temperature						
Mean	−3.90	−4.37	−3.90	−2.94	−2.02	−2.56	−1.90	−1.07	3.39	2.90	3.47	4.30
Min.	−29.5	−29.5	−21.1	−19.0	−19.5	−19.5	−17.3	−14.6	−11.6	−11.6	−11.6	−7.96
Max	10.3	9.11	8.49	10.3	11.6	10.4	9.98	11.6	15.6	14.1	13.9	15.6
Skewness	−0.16	−0.21	−0.12	−0.05	−0.14	−0.15	−0.17	−0.06	−0.16	−0.18	−0.22	−0.06
Std. dev.	8.76	8.91	8.76	8.35	8.15	8.19	8.22	7.92	7.18	7.20	7.22	7.02
				Extraterrestrial radiation								
Mean	30.2	30.2	30.2	30.2	30.2	30.2	30.2	30.2	30.6	30.6	30.6	30.6
Min.	17.3	17.3	17.3	17.3	17.4	17.4	17.4	17.4	18.1	18.1	18.1	18.1
Max	41.5	41.5	41.5	41.5	41.5	41.5	41.5	41.5	41.4	41.4	41.4	41.4
Skewness	−0.13	−0.13	−0.13	−0.13	−0.14	−0.14	−0.14	−0.14	−0.15	−0.15	−0.15	−0.15
Std. dev.	8.62	8.62	8.62	8.62	8.60	8.60	8.60	8.60	8.33	8.33	8.33	8.33
						Evaporation						
Mean	2.38	2.34	2.42	2.40	2.28	2.27	2.28	2.29	2.43	2.40	2.40	2.50
Min.	0.33	0.33	0.52	0.63	0.57	0.57	0.61	0.73	0.65	0.71	0.65	0.78
Max	4.93	4.36	4.93	4.73	4.58	4.58	4.30	4.40	4.64	4.60	4.35	4.64
Skewness	0.08	0.03	0.18	0.07	0.07	0.09	0.03	0.05	0.01	0.01	0.02	0.01
Std. dev.	1.10	1.11	1.16	1.03	1.07	1.08	1.07	1.02	1.07	1.07	1.10	1.06

Table S2. Validation and test statistics of the models for monthly ET0 prediction – Station 1 (56004).

Model Inputs	Validation Period			Test Period		
	RMSE	MAE	R^2	RMSE	MAE	R^2
			LSSVM-GSA			
T_t	0.422	0.310	0.872	0.420	0.320	0.856
T_t, T_{t-1}	0.260	0.190	0.944	0.252	0.186	0.949
T_t, T_{t-1}, T_{t-2}	0.246	0.184	0.958	**0.246**	**0.182**	**0.953**
T_t, T_{t-1}, T_{t-2}, T_{t-3}	0.265	0.193	0.950	0.248	0.188	0.951
Ra_t	0.338	0.244	0.921	0.285	0.227	0.932
Ra_t, Ra_{t-1}	0.340	0.249	0.920	0.282	0.223	0.934
Ra_t, Ra_{t-1}, Ra_{t-2}	0.341	0.246	0.920	0.279	0.220	0.934
Ra_t, Ra_{t-1}, Ra_{t-2}, Ra_{t-3}	0.341	0.246	0.920	**0.277**	**0.219**	**0.935**
			DENFIS			
T_t	0.427	0.315	0.866	0.424	0.327	0.851
T_t, T_{t-1}	0.264	0.195	0.949	0.255	0.188	0.947
T_t, T_{t-1}, T_{t-2}	0.256	0.194	0.952	0.258	0.194	0.950
T_t, T_{t-1}, T_{t-2}, T_{t-3}	0.271	0.198	0.947	**0.249**	**0.190**	**0.950**
Ra_t	0.373	0.289	0.903	0.308	0.243	0.919
Ra_t, Ra_{t-1}	0.346	0.252	0.919	0.290	0.230	0.932
Ra_t, Ra_{t-1}, Ra_{t-2}	0.344	0.249	0.918	0.285	0.223	0.934
Ra_t, Ra_{t-1}, Ra_{t-2}, Ra_{t-3}	0.342	0.248	0.919	**0.281**	**0.221**	**0.934**
			M5RT			
T_t	0.451	0.310	0.849	0.445	0.335	0.832
T_t, T_{t-1}	0.319	0.226	0.925	0.330	0.244	0.915
T_t, T_{t-1}, T_{t-2}	0.322	0.221	0.923	**0.305**	**0.232**	**0.928**
T_t, T_{t-1}, T_{t-2}, T_{t-3}	0.343	0.246	0.913	0.315	0.234	0.916
Ra_t	0.378	0.307	0.899	0.352	0.293	0.917
Ra_t, Ra_{t-1}	0.350	0.266	0.917	**0.323**	**0.267**	**0.932**
Ra_t, Ra_{t-1}, Ra_{t-2}	0.351	0.264	0.916	0.328	0.269	0.931

Table S3. Validation and test statistics of the models for monthly ET0 prediction – Station 2 (56021).

Model Inputs	Validation Period			Test Period		
	RMSE	MAE	R^2	RMSE	MAE	R^2
	LSSVM-GSA					
T_t	0.390	0.306	0.871	0.454	0.383	0.855
T_t, T_{t-1}	0.245	0.170	0.952	0.255	0.205	0.942
T_t, T_{t-1}, T_{t-2}	0.235	0.161	0.954	**0.230**	**0.179**	**0.956**
T_t, T_{t-1}, T_{t-2}, T_{t-3}	0.241	0.170	0.952	0.234	0.183	0.953
Ra_t	0.304	0.223	0.919	0.267	0.203	0.934
Ra_t, Ra_{t-1}	0.277	0.198	0.933	0.242	0.180	0.945
Ra_t, Ra_{t-1}, Ra_{t-2}	0.275	0.197	0.934	0.237	0.178	0.948
Ra_t, Ra_{t-1}, Ra_{t-2}, Ra_{t-3}	0.276	0.195	0.933	**0.236**	**0.176**	**0.949**
	DENFIS					
T_t	0.393	0.297	0.872	0.443	0.352	0.840
T_t, T_{t-1}	0.249	0.171	0.946	0.326	0.263	0.936
T_t, T_{t-1}, T_{t-2}	0.245	0.168	0.949	0.320	0.264	0.943
T_t, T_{t-1}, T_{t-2}, T_{t-3}	0.242	0.162	0.951	**0.301**	**0.253**	**0.945**
Ra_t	0.311	0.229	0.920	0.286	0.225	0.933
Ra_t, Ra_{t-1}	0.291	0.212	0.928	0.265	0.204	0.941
Ra_t, Ra_{t-1}, Ra_{t-2}	0.287	0.203	0.931	0.262	0.203	0.943
Ra_t, Ra_{t-1}, Ra_{t-2}, Ra_{t-3}	0.286	0.209	0.932	**0.241**	**0.186**	**0.947**
	M5RT					
T_t	0.442	0.332	0.838	0.483	0.373	0.803
T_t, T_{t-1}	0.329	0.233	0.916	0.433	0.315	0.865
T_t, T_{t-1}, T_{t-2}	0.265	0.189	0.940	**0.310**	**0.227**	**0.921**
T_t, T_{t-1}, T_{t-2}, T_{t-3}	0.315	0.233	0.920	0.349	0.262	0.907
Ra_t	0.299	0.221	0.912	0.271	0.204	0.924
Ra_t, Ra_{t-1}	0.294	0.215	0.916	0.267	0.199	0.929
Ra_t, Ra_{t-1}, Ra_{t-2}	0.289	0.211	0.929	**0.251**	**0.191**	**0.942**
Ra_t, Ra_{t-1}, Ra_{t-2}, Ra_{t-3}	0.292	0.213	0.925	0.256	0.194	0.940

Table S4. Validation and test statistics of the models for monthly ET0 prediction – Station 3 (56029).

Model Inputs	Validation Period			Test Period		
	RMSE	MAE	R^2	RMSE	MAE	R^2
	LSSVM-GSA					
T_t	0.459	0.362	0.833	0.472	0.395	0.811
T_t, T_{t-1}	0.248	0.195	0.957	0.319	0.240	0.944
T_t, T_{t-1}, T_{t-2}	0.209	0.163	0.967	0.233	0.176	0.953
T_t, T_{t-1}, T_{t-2}, T_{t-3}	0.202	0.160	0.968	**0.230**	**0.172**	**0.954**
Ra_t	0.234	0.184	0.955	0.262	0.207	0.945
Ra_t, Ra_{t-1}	0.235	0.181	0.956	0.266	0.205	0.945
Ra_t, Ra_{t-1}, Ra_{t-2}	0.230	0.178	0.957	0.264	0.207	0.946
Ra_t, Ra_{t-1}, Ra_{t-2}, Ra_{t-3}	0.230	0.179	0.957	**0.262**	**0.205**	**0.947**
	DENFIS					
T_t	0.464	0.357	0.830	0.497	0.412	0.828
T_t, T_{t-1}	0.253	0.192	0.960	0.340	0.275	0.938
T_t, T_{t-1}, T_{t-2}	0.212	0.161	0.966	0.309	0.256	0.950
T_t, T_{t-1}, T_{t-2}, T_{t-3}	0.217	0.160	0.964	**0.291**	**0.239**	**0.951**

Ra_t	0.231	0.178	0.957	**0.268**	**0.209**	**0.944**
Ra_t, Ra_{t-1}	0.235	0.182	0.955	0.272	0.213	0.942
Ra_t, Ra_{t-1}, Ra_{t-2}	0.233	0.180	0.956	0.270	0.212	0.943
Ra_t, Ra_{t-1}, Ra_{t-2}, Ra_{t-3}	0.232	0.180	0.956	0.269	0.210	0.943
			M5RT			
T_t	0.530	0.375	0.777	0.551	0.416	0.740
T_t, T_{t-1}	0.293	0.222	0.933	0.341	0.254	0.909
T_t, T_{t-1}, T_{t-2}	0.300	0.224	0.929	0.361	0.276	0.900
T_t, T_{t-1}, T_{t-2}, T_{t-3}	0.276	0.209	0.938	**0.317**	**0.224**	**0.910**
Ra_t	0.241	0.197	0.952	0.276	0.229	0.933
Ra_t, Ra_{t-1}	0.239	0.195	0.953	0.274	0.219	0.935
Ra_t, Ra_{t-1}, Ra_{t-2}	0.248	0.199	0.951	0.273	0.218	0.937
Ra_t, Ra_{t-1}, Ra_{t-2}, Ra_{t-3}	0.232	0.193	0.954	**0.272**	**0.215**	**0.940**
